# Stem girdling evidences a trade-off between cambial activity and sprouting and dramatically reduces plant transpiration due to feedback inhibition of photosynthesis and hormone signaling

**DOI:** 10.3389/fpls.2015.00285

**Published:** 2015-04-29

**Authors:** Rosana López, Ricard Brossa, Luis Gil, Pilar Pita

**Affiliations:** ^1^Forest Genetics and Physiology Research Group, School of Forest Engineering, Technical University of MadridMadrid, Spain; ^2^Department of Plant Biology, Faculty of Biology, University of BarcelonaBarcelona, Spain

**Keywords:** sprouting, source–sink regulation, gas exchange, chlorophyll fluorescence, hydraulic conductance, ABA

## Abstract

The photosynthesis source–sink relationship in young *Pinus canariensis* seedlings was modified by stem girdling to investigate sprouting and cambial activity, feedback inhibition of photosynthesis, and stem and root hydraulic capacity. Removal of bark tissue showed a trade-off between sprouting and diameter growth. Above the girdle, growth was accelerated but the number of sprouts was almost negligible, whereas below the girdle the response was reversed. Girdling resulted in a sharp decrease in whole plant transpiration and root hydraulic conductance. The reduction of leaf area after girdling was strengthened by the high levels of abscisic acid found in buds which pointed to stronger bud dormancy, preventing a new needle flush. Accumulation of sugars in leaves led to a coordinated reduction in net photosynthesis (A_N_) and stomatal conductance (g_S_) in the short term, but later (g_S_ below 0.07 mol m^-2^ s^-1^) A_N_ decreased faster. The decrease in maximal efficiency of photosystem II (F_V_/F_M_) and the operating quantum efficiency of photosystem II (ΦPSII) in girdled plants could suggest photoprotection of leaves, as shown by the vigorous recovery of A_N_ and ΦPSII after reconnection of the phloem. Stem girdling did not affect xylem embolism but increased stem hydraulic conductance above the girdle. This study shows that stem girdling affects not only the carbon balance, but also the water status of the plant.

## Introduction

Photosynthesis products are modulated by source–sink equilibria within the plant (Jang and Sheen, 1994) and hydraulic constraints (Brodribb and Field, 2000) including the activity of root meristems. Phloem serves as the long-distance transport pathway for photosynthate movement from source leaves to regions of active growth, storage structures, and other non-photosynthetic cells. Natural loss of phloem and bark can occur after severe perturbations such as wounding caused by volcanic activity (Rodríguez Martín et al., 2013) or when small rodents chew away the bark down to the cambium layer of seedlings during winter. Artificial girdling has traditionally been used to study apical control of branch growth (Münch, 1938) and the response dynamics of phloem transport (Parker, 1974), and to assess the effect of ion redistribution between phloem and xylem on xylem flow rate (Zwieniecki et al., 2004). In horticulture, girdling has been widely applied to increase flowering, fruit set and fruit size (Williams et al., 2000; Di Vaio et al., 2001; Williams and Ayars, 2005; Rivas et al., 2007; Urban and Alphonsout, 2007) and in forestry it has been used to alter wood properties (Noel, 1970). The effect of stem girdling at the base of the stem differs from the effect of branch girdling commonly used in agricultural practices, because besides promoting carbohydrate accumulation above the girdle it prevents phloem transport to the roots. Despite its ecological significance, studies about the physiological effects of complete stem girdling in forest trees are scarce compared to those carried out on branch girdling in horticulture.

**FIGURE 1:**
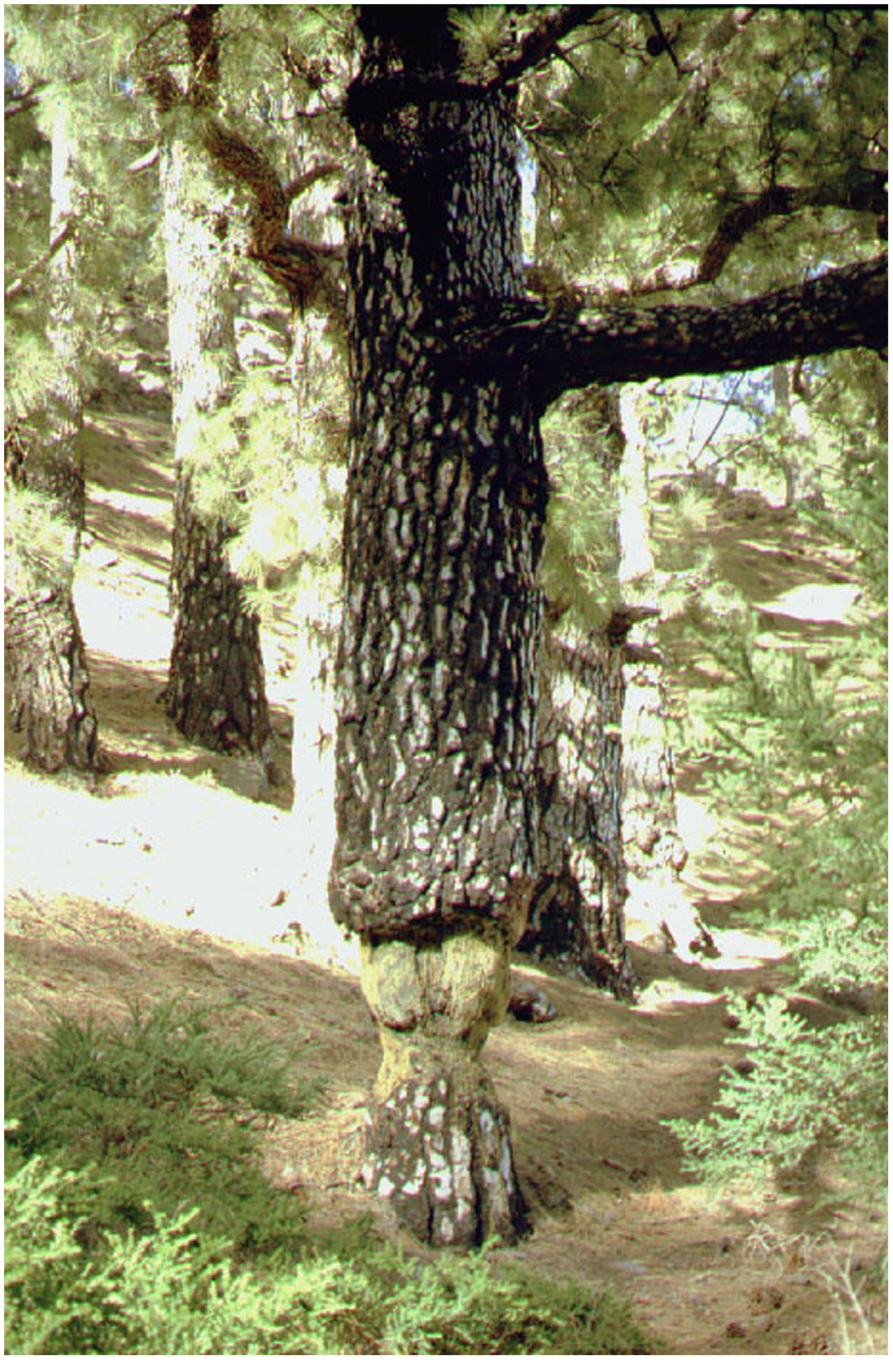
***Pinus canariensis* showing severe girdling (10 cm wide), with a small strip of phloem 3 cm wide.** The tree was 65 years old when girdled and had a diameter of 56 cm. After 55 years, diameter increment was 1 cm below the girdle, compared to 30 cm above.

The Canary Island pine (*Pinus canariensis*) is endemic to the Canary Islands with a broader distribution in the past, when volcanism represented a strong selection factor (Frankis, 1999). It is one of the few pines able to resprout after severe injury, a trait traditionally associated with fire resilience (Climent et al., 2004), and it is also capable of surviving after partial girdling of the stem, with only a small strip of phloem 3 cm wide, for more than 60 years (**Figure 1**).

The first visual symptom after girdling is an increase in stem diameter above the girdle due to growth of bark and xylem stimulated by the accumulation of soluble sugars and starch, coupled with the interruption of stem growth below the girdle (Daudet et al., 2005; De Schepper et al., 2010). Roots are gradually depleted of their carbohydrate reserves and can suffer starvation (Weaver and McCune, 1959). Accumulation of carbohydrates above the girdle enhances the respiratory potential of the stem and branches and also increases leaf mass per area (LMA; Domec and Pruyn, 2008). This stock of sugars has been used to explain the decline in leaf photosynthesis due to end-product inhibition (Iglesias et al., 2002) and the reduction in quantum yield efficiency of photosystem II through feedback loops (Jang et al., 1997; Myers et al., 1999; Rivas et al., 2007; Urban and Alphonsout, 2007; Murakami et al., 2008). At the same time, removal of the sink demand of roots generally results in decreased stomatal conductance and transpiration, accumulation of abscisic acid (ABA) in leaves (Setter et al., 1980), and an increase in leaf water potential (Williams et al., 2000).

Although some studies assumed that girdling affects only carbon status and not water status (e.g., Daudet et al., 2005; Murakami et al., 2008), there is evidence that disruption of phloem transport reduces xylem conductivity and embolism repair, at least in some species (Salleo et al., 1996; Zwieniecki and Holbrook, 1998; Christman et al., 2012). The partial closure of stomata in girdled trees leads to a reduction in leaf hydraulic conductance (Domec and Pruyn, 2008; Sellin et al., 2013) resulting from regulation of membrane aquaporins (Kuwagata et al., 2012) or even occlusion of leaf veins (Sellin et al., 2013), and also to a decline in branch hydraulic capacity following changes in the sapwood to leaf area ratio (Domec and Pruyn, 2008). The long-term effect, even when the phloem has reconnected, entails changes in cambial activity in the following years. In conifers, tracheids are shorter and wider and more efficient in water transport but less resistant to cavitation in girdled trees (Domec and Pruyn, 2008). Girdling changes the properties of sapwood, particularly below the girdle, where stem moisture content and parenchyma viability are significantly reduced (Taylor and Cooper, 2002).

Blocking the downward translocation of photosynthates and metabolites through the phloem can also affect leaf size and number (Fumuro, 1998) and promote sprouting (De Schepper et al., 2010). To our knowledge, the effect on the potential trade-offs between sprouting and cambial activity as carbohydrate sinks has never been assessed. Sprouting as a means of persistence is thought to be strongly linked to the frequency, severity and predictability of disturbances and how these disturbances interact with regrowth rates (Bond and Midgley, 2003). Sprouter species from fire-prone ecosystems generally allocate more carbon to starch or other reserves in the roots than non-sprouters (Bell and Ojeda, 1999). Thus the effect of girdling on roots and radial growth could be lessened in these species. Sprouting is a ubiquitous feature of temperate angiosperm trees, at least in saplings (Del Tredici, 2001), but among conifers this ability is restricted in adults to a few species (Keeley and Zedler, 1998; Del Tredici, 2001).

In this study we monitor whole-plant physiological responses to girdling in 3-years-old seedlings of Canary Island pine to identify potential tradeoffs between hydraulic properties and photosynthesis and their influence in growth and aboveground biomass production. Two girdling treatments were tested to assess the impact of phloem reconnection in some plants. We hypothesized that (i) sprouting would be profuse after girdling, affecting radial growth of the stem (ii) root depletion of carbohydrates would decrease hydraulic conductivity, affecting leaf water status, triggering stomatal closure and eventually damaging the photosynthetic apparatus, and (iii) carbon starvation in roots would increase ABA synthesis promoting leaf senescence via hormone signaling.

## Materials and Methods

### Plant Material and Phloem Girdling

**FIGURE 2:**
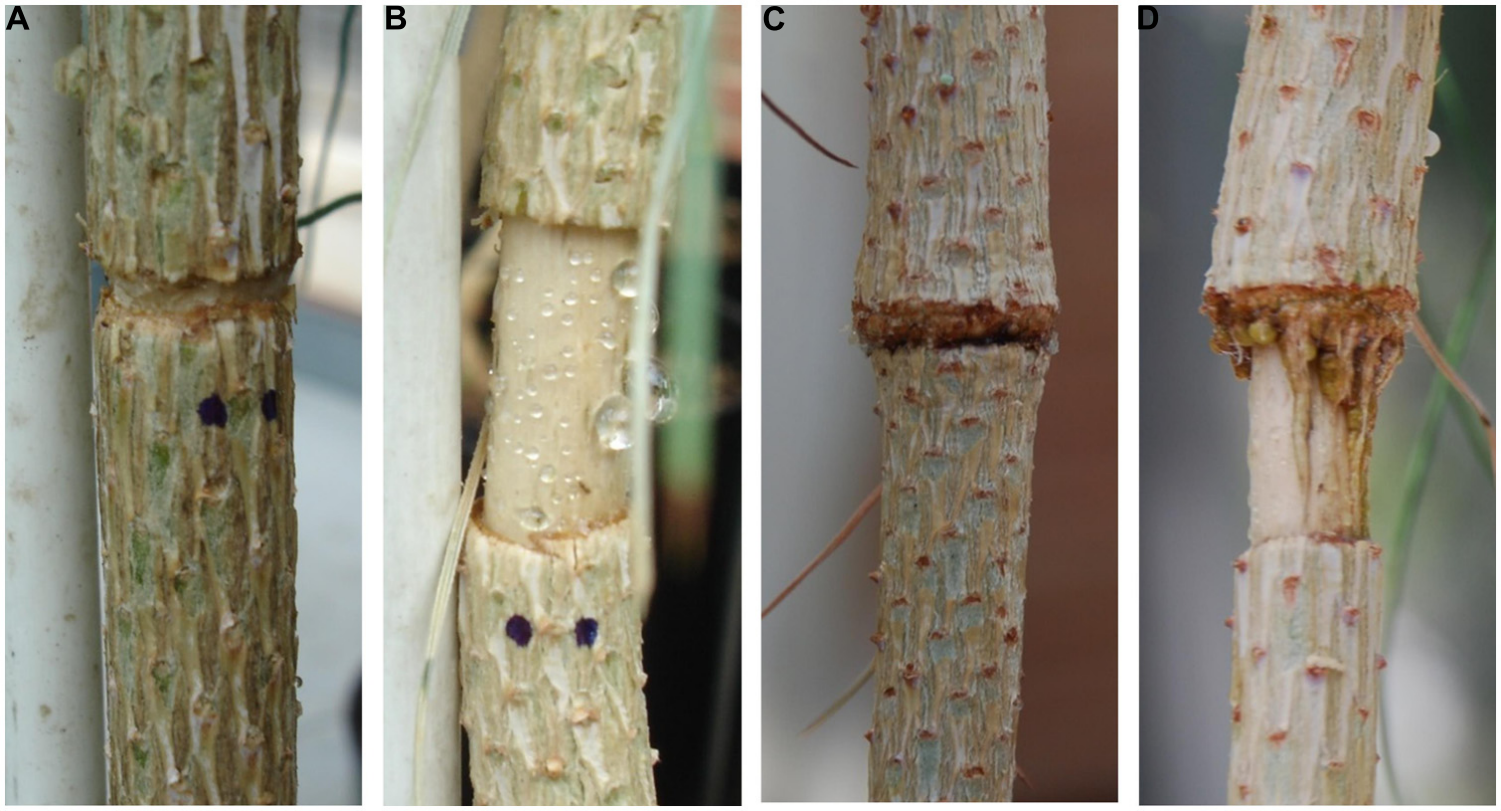
**Girdling treatments: **(A)** fine girdling (GF) and **(B)** wide girdling (GW) 1 week after girdling.** Formation of wound tissue on sites where bark tissue was removed 50 days after girdling (d.a.g.): **(C)** reconnection of the phloem in GF and **(D)** vertical strands from the top of the girdle in GW growing over to reconnect the two sides of the girdle.

A total of 60 3-years-old *P. canariensis* trees were grown in a greenhouse in 5 L pots filled with 1/4 perlite and 3/4 peat, v:v, and fertilized with a controlled release fertilizer (Nutricote, Chisso Asahi Fertilizer, Japan). At the beginning of May 2011, the plants were distributed in three blocks according to size. Average tree height in each block was 105, 80, and 70 cm and average basal diameter (i.e., at ground level) was 9.4, 9.2, and 7.0 cm, respectively. Two girdling treatments were applied in each block: fine girdling, (GF, 3 mm strip of bark, five plants per block) and wide girdling (GW, 15 mm strip of bark, 10 plants per block) and remaining plants were used as controls (C, five plants per block). In the girdled trees, a strip of bark (periderm, cortex, and phloem) was removed by inserting a razor blade at 1/3 height from the base (**Figures 2A,B**). All needles below the wound were removed.

### Radial Growth and Aboveground Biomass

Stem diameter variations above and below the girdle (U and L, respectively) and in the base of the stem (B) were measured throughout the experiment with a digital caliper. The number of sprouts in U, L, and B was counted and sprouts were removed periodically after girdling to avoid new sources of carbohydrates below the girdle. Aboveground dry biomass was measured at the beginning of July (56 days after girdling, from now d.a.g.) in all plants from block 1 and at the end of the experiment (145 d.a.g.) in plants from blocks 2 and 3. After plant harvest, needles were detached and the stem was used for hydraulic conductance measurements. Stem and needle dry weights were measured after drying to constant weight at 80°C.

### Plant Transpiration and Hydraulic Conductance

Whole plant transpiration was estimated gravimetrically by measuring the loss of mass from the pot every two-3 days before watering to field capacity in two phases. The first phase was from the beginning of the experiment until the first harvest (56 d.a.g.) and the second was from 112 d.a.g. to the final harvest (145 d.a.g.). Plants were kept well-watered throughout the experiment. Leaf area specific transpiration for each block and treatment was calculated as the maximum transpiration in the week previous to harvest per unit of projected leaf area.

Just after the first harvest, stem hydraulic conductance (k_h_) and native embolism, i.e., initial embolism naturally occurring or caused by the girdling treatment, were measured in two segments in L and in three segments in U with a XYL’EM (Xylem Embolism Meter, Bronkhorst, Montigny les Cormeilles, France). All segments were perfused with degassed and filtered (0.2 μm) 10 mM KCl and 1 mM CaCl_2_ solution in ultrapure water. Initial conductance (k_in_) was measured at low pressure (6 kPa) after the stems were cut under water. Embolism was removed at high pressure (0.18 MPa) for 20 min and maximum hydraulic conductance (k_max_) was determined. For each segment, stem specific conductivity (K_S_) was calculated by multiplying k_max_ by sample length and dividing by xylem cross section and leaf-specific hydraulic conductivity (LSC), as the conductivity per unit of projected leaf area.

Whole root system conductance (k_root_) was measured on severed root systems kept in their substrate, with the stump connected to a high pressure flow meter (HPFM, Dynamax Inc., Houston, TX, USA). Shoots were excised about 1 cm above the root collar and attached to the HPFM. Measurements were made in the transient mode. During each measurement the pressure was increased from 0.03 to 0.45 MPa at a rate of 5–8 KPa s^-1^ and the flow rate was recorded every 3 s. k_root_ was calculated from the linear region of the relationship between flow rate and pressure (Tyree et al., 1995). Two to eight measurements were made in each plant. We did not observe any pattern of decrease/increase in k_root_ with measurements. We therefore hypothesized that any change in k_root_ between measurements made on a single plant was caused by partial tracheid blockage with resin. Leaf-specific root hydraulic conductance (LSc_root_) was calculated as the root conductance per unit of projected leaf area.

### Wood Density

Wood density was determined on 2.5 cm-long segments above and below the girdle. Green volume of the wood sample was determined according to Archimedes’ principle. Samples were stored at 105°C for 48 h and their dry weight was measured. Basic density was calculated as the ratio of dry weight to green volume.

### Gas Exchange and Chlorophyll Fluorescence

Simultaneous measurements of gas exchange and chlorophyll fluorescence were made with an Li-6400 open gas exchange system (Li-Cor, Lincoln, NE, USA) and with an Li-6400-40 integrated fluorescence chamber (Li-Cor, Lincoln, NE, USA) on samples of nine fully expanded adult needles. Measurements were made in May, June, and September. Each block was measured on consecutive days (18, 19, 20 d.a.g., 35, 36, 37 d.a.g., and 140, 141 d.a.g.). All surviving plants were measured each time. As one block was harvested in July (56 d.a.g.), only two blocks were measured in September. Gas exchange parameters – net photosynthesis (A_N_), stomatal conductance (g_S_), transpiration rate (E), and leaf temperature (T_L_) – were measured with saturating light at ambient CO_2_ and ambient temperature. Steady state fluorescence (F_S_) and maximum fluorescence from a light adapted sample (F_M_’) were calculated after saturation flash (8000 μmol m^-2^ s^-1^, 0.8 s). The operating quantum efficiency of photosystem II (ΦPSII) was calculated as ΦPSII = 1 – F_S_/F_M_’ (Genty et al., 1989).

Chlorophyll fluorescence was measured separately from gas exchange, on the same plants and 2–3 days after the other measurements, from 9:00 to 11:30 h, on nine fully expanded adult needles after at least 40 min of dark adaptation. The ratio of variable to maximum chlorophyll fluorescence (F_V_/F_M_) was calculated as (F_M_ – F_0_)/F_M_, where F_M_ and F_0_ were, respectively, the maximum and basal fluorescence yields of dark-adapted needles. This parameter provides an estimate of the maximum photochemical efficiency of photosystem II.

### Chlorophyll Content, Leaf Mass per Area and Leaf Relative Water Content

Three times during the experiment, at 35, 50, and 140 d.a.g., chlorophyll a and b and carotenoid concentrations in three needles per plant were determined spectrophotometrically after extraction with dimethyl sulfoxide at 60°C for 4 h, following Wellburn (1994).

At the end of the experiment, LMA was measured in 18 needles from each sampled tree. Needle projected area was obtained with a scanner and the images were analyzed with WinFOLIA (Regent Instruments). Needles were dried at 80°C for 2 days to determine leaf dry mass and LMA. In another subsample of needles, relative water content (RWC) was determined as: RWC = (FW – DW)/(TW – DW), where FW is fresh weight, TW is turgid weight after rehydrating needles for 24 h at 4°C in darkness, and DW is dry weight after oven-drying needles for 48 h at 80°C.

### Hormone Concentration

Concentrations of ABA, abscisic acid glucose ester (ABA-GE), and jasmonic acid (JA) in xylem, needles, buds, and phloem above and below the girdle were simultaneously analyzed by HPLC MS/MS, as described by Brossa et al. (2011). 100 mg fresh tissue was ground in liquid nitrogen with a mortar and pestle and extracted with 750 μl methanol–water–acetic acid (90:9:1 v/v/v). Deuterium-labeled internal standards (40 ng ABA-d_6_, 40 ng ABA-GE-d_2_, and 40 ng JA-d_5_) were added to each sample at the beginning of the extraction procedure. Extracts were vortexed for 5 min and incubated for 10 min at 4°C under ultrasonication (Vibra-Cell Ultrasonic Processor, Sonics & Materials Inc., Newtown, CT, USA) then centrifuged for 10 min at 10,000 rpm. The supernatants were collected and the pellets were re-extracted with 750 μl of the extraction solvent. Pellets were then pooled and filtered. 5 μl of each sample was injected into the LC system (Acquity UPLC, Waters) using a Waters X-Bridge C18 column (3.5 μm; 100 9 2.1 i.d.). The MS/MS quantification was performed on an API 3000 triple quadrupole mass spectrometer (AB Sciex, Danaher Corp., Washington, DC, USA) using multiple reaction monitoring (MRM) acquisition with the corresponding transitions for each analyte.

### Statistical Analysis

The effects of *treatment*, *block* and *position in the stem* (U, L, and B) on diameter and number of sprouts were checked using repeated measures analyses of variance (ANOVA). Repeated measures ANOVA were also run to analyze transpiration, gas exchange, chlorophyll fluorescence, and chlorophyll content. Wood density was analyzed using a GLM approach to ANOVA with the factors *treatment* and *block*. One-way ANOVA with *treatment* as the only factor was performed on aboveground biomass, LMA, RWC, hydraulic conductance parameters, and hormone content. All factors were considered fixed for all the analyses. Differences between levels of significant predictors were tested by Duncan’s multiple-range tests.

Pearson’s product-moment correlation coefficients were calculated to relate gas exchange, chlorophyll fluorescence and growth traits. Bivariate relationships between the traits studied and independent variables were assessed using simple linear or non-linear least squares regression.

## Results

### Stem Diameter Variations and Sprouting

**FIGURE 3:**
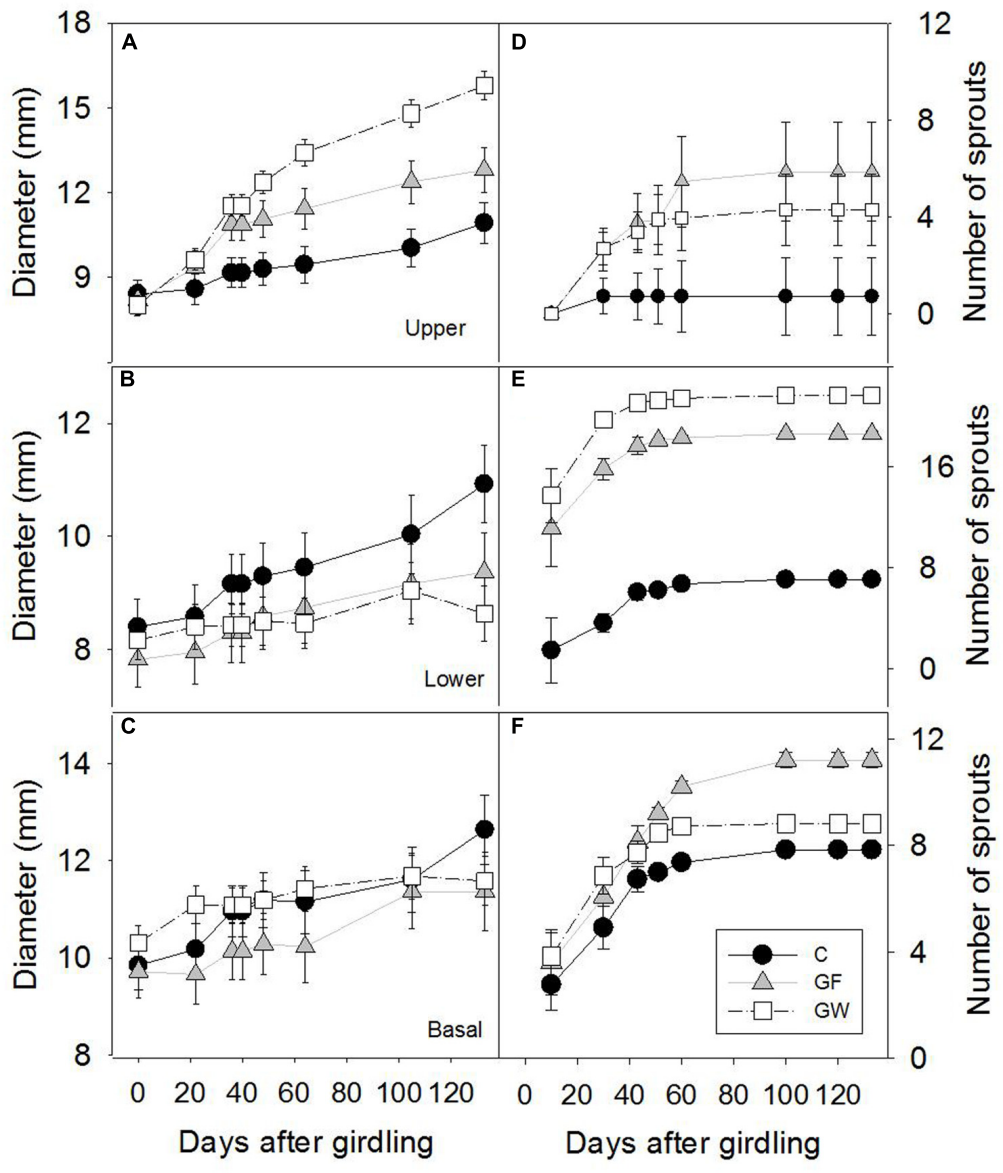
**Time frame of stem diameter growth **(A)** above the girdle (U), **(B)** below the girdle (L) and **(C)** in the base of the stem (B) and accumulated number of sprouts **(D)** above the girdle, **(E)** below the girdle and **(F)** in the base of the stem in control (C), in fine girdled (GF) and wide girdled (GW) plants.** Error bars represent the standard error.

Girdling resulted in immediate reduction in radial growth of the segment below the girdle (L) and later of the base of the stem (B), whereas growth of the upper segment (U) increased significantly (**Figure 3**). Approximately 48 d.a.g., coinciding with the apparent reconnection between U and L in most plants with fine girdling (GF), the rate of radial growth of U in GF decreased, and after this date, the upper segment of plants with wide girdling (GW) was significantly wider than in GF (*p* < 0.001; **Figure 3A**).

**FIGURE 4:**
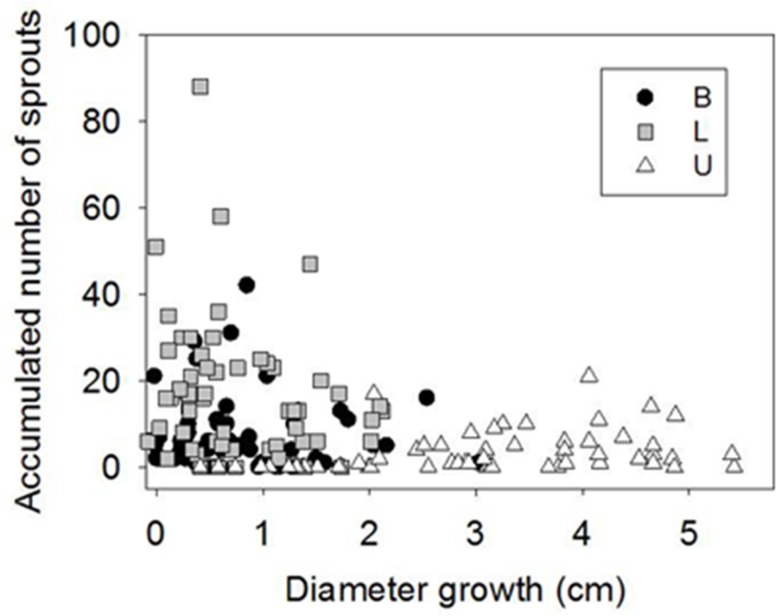
**Diameter growth vs. accumulated number of sprouts in control and girdled plants in the base of the stem (B), below the girdle (L) and above the girdle (U) 48 d.a.g**.

Sprouting was profuse after girdling during the first month of the experiment, but from 50 d.a.g. it virtually ceased. The number of sprouts in B and L was much higher than in U (**Figures 3D–F**). In general, profuse sprouting was associated with lower diameter growth (**Figure 4**). Total leaf area and aboveground dry mass were higher in C than in GF and GW in the first harvest but differed significantly only between C and GW in the final harvest (**Table 1**). At the end of the experiment, LMA in GW was higher and RWC was lower than in C and GF (**Table 1**).

**Table 1:** Mean values (±SE) for aboveground dry mass, leaf area, and maximum leaf specific transpiration measured 56 days after girdling (d.a.g.) and at the end of the experiment (145 d.a.g.), leaf mass per area and relative water content of control (C) and girdled *Pinus canariensis*.

	C	GF	GW
Dry mass (g) 56 d.a.g.	75.8 ± 7.1^a^	49.0 ± 6.9^b^	54.0 ± 5.2^b^
Dry mass (g) 145 d.a.g. (block 2/3)	70.7 ± 5.5^a^/49.8 ± 3.1^b^	60.6 ± 6.5^ab^/44.9 ± 4.7^b^	56.2 ± 4.4^ab^/27.0 ± 6.3^c^
Leaf area (m^2^) 56 d.a.g.	0.51 ± 0.05^a^	0.36 ± 0.05^b^	0.33 ± 0.03^b^
Leaf area (m^2^) 145 d.a.g. (block 2/3)	0.35 ± 0.04^a^/0.31 ± 0.04^a^	0.33 ± 0.03^a^/0.32 ± 0.04^a^	0.26 ± 0.03^b^/0.11 ± 0.04^c^
Leaf specific trans (g m^-2^ day^-1^) 56 d.a.g. (block 1)	797 ± 85^a^	670 ± 46^a^	764 ± 63^a^
Leaf specific trans (g m^-2^ day^-1^) 145 d.a.g. (block 2/3)	763 ± 79^a^/855 ± 56^a^	532 ± 58^b^/651 ± 76^ab^	197 ± 29^c^/210 ± 46^c^
LMA (g m^-2^)145 d.a.g.	115.4 ± 4.0^b^	115.5 ± 4.5^b^	126.7 ± 4.1^a^
RWC 145 d.a.g.	0.95 ± 0.05^a^	0.96 ± 0.05^a^	0.79 ± 0.03^b^

GF, fine girdled; GW, wide girdled (see Materials and Methods). Identical letters indicate homogeneous groups (*p* < 0.05).

**FIGURE 5:**
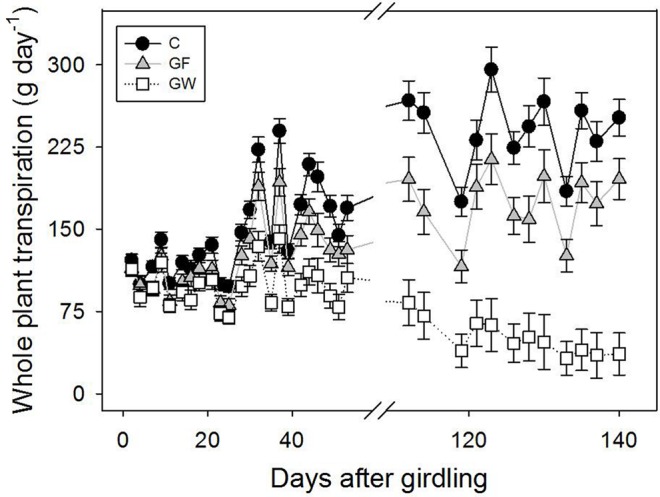
**Changes in whole plant transpiration in control (C), fine girdled (GF), and wide girdled (GW) plants.** Reconnection of the phloem in GF was ca. 48 d.a.g. Error bars represent the standard error.

Transpiration was lower in girdled than in control plants throughout the experiment but differences were considerable in the second phase, after the warmest months, when water loss in GW was four times lower than in C. Although in this second phase most GF had apparently reconnected the vascular tissue, transpiration did not fully recover and was 25% lower than in C (**Figure 5**), despite the lack of differences in leaf area between C and GF (**Table 1**). When considering leaf area specific transpiration, differences between treatments were not significant in the first phase of the experiment, whereas in the second phase the whole plant transpiration pattern was confirmed (**Table 1**).

### Gas Exchange and Chlorophyll Fluorescence Parameters

**FIGURE 6:**
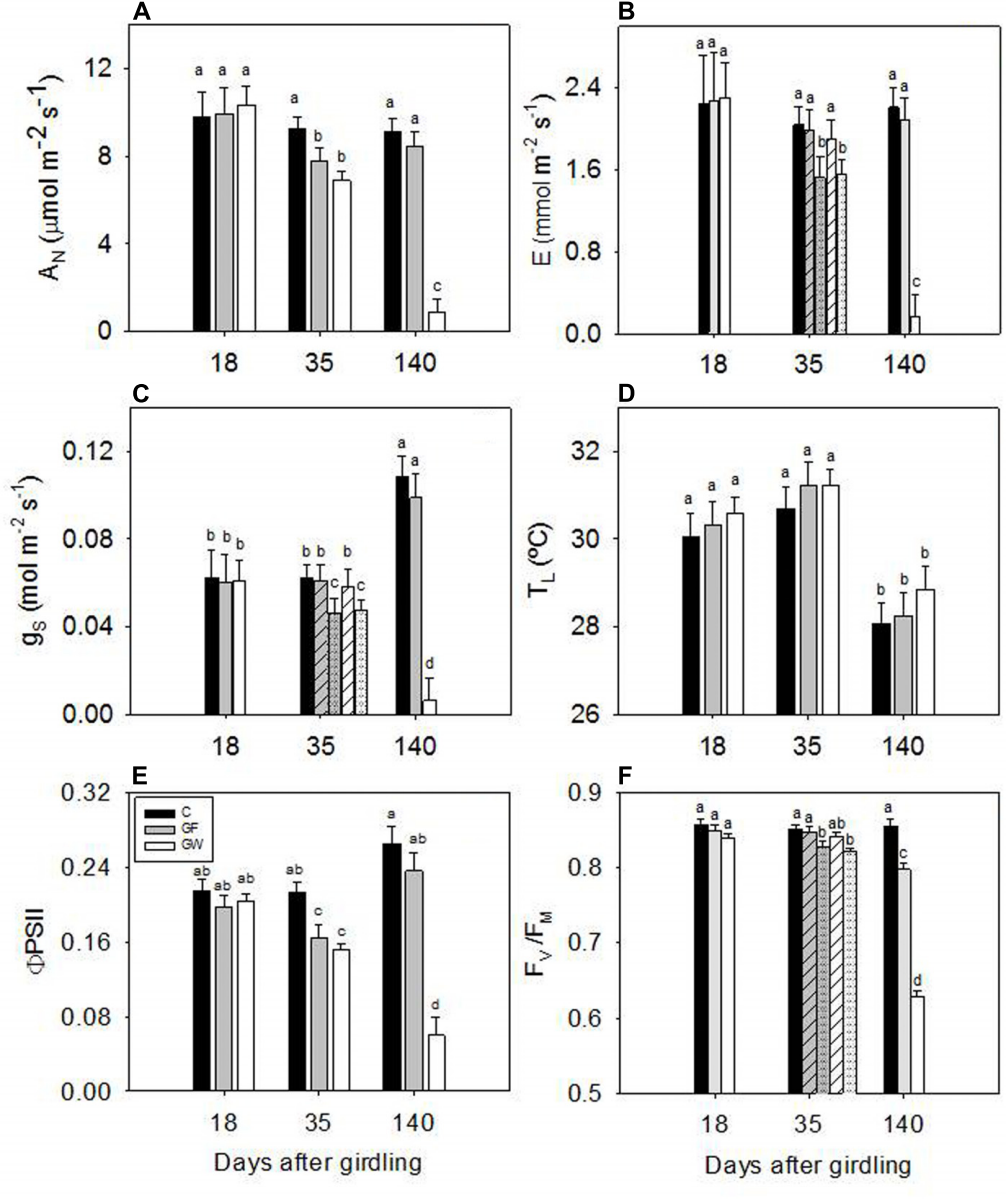
**Changes in **(A)** net photosynthesis (A_N_), **(B)** transpiration rate (E), **(C)** stomatal conductance to water vapor (g_S_), **(D)** leaf temperature (T_L_), **(E)** operating quantum efficiency of photosystem II electron transport (ΦPSII) and **(F)** maximum photochemical efficiency of photosystem II in dark adapted leaves (F_V_/F_M_) in response to phloem girdling.** Error bars represent the standard error and significant differences (*p* < 0.05) are indicated by different letters. Differences between blocks 35 d.a.g. in E, g_S_ and F_V_/F_M_ in girdled plants are shown with striped bars for larger plants (block 1) and dotted bars for plants in blocks 2 and 3.

Gas exchange and chlorophyll fluorescence values 18 d.a.g. were similar among treatments. On following dates, net photosynthesis (A_N_), transpiration (E) and stomatal conductance (g_S_) decreased strongly in GW and 140 d.a.g. gas exchange values were more than 10 times lower than in C and GF (**Figures 6A–C**). ΦPSII decreased steadily in GW, reaching a value of 0.05 at the end of the experiment, five times lower than in C and GF (**Figure 6E**). At 35 d.a.g. A_N_ differed between girdled and control plants, whereas we found significant differences in g_S_ and E between blocks in girdled plants: larger plants did not differ from C whereas smaller plants showed lower values (**Figure 6B,C**). At the end of the experiment, values in GF recovered to C values. F_V_/F_M_ remained almost constant at 0.85 in C throughout the experiment. At 35 d.a.g. we again found a block effect in girdled plants: F_V_/F_M_ of larger plants was 0.85 but in smaller plants it decreased to 0.82. At 140 d.a.g. F_V_/F_M_ almost recovered to C values in GF, but GW values dropped to 0.63 (**Figure 6F**). At 35 d.a.g. chlorophyll content and carotenoids in both GW and GF were lower than in C. However, measurements made 50 and 140 d.a.g. showed that photosynthetic pigment content of GF had recovered and differed significantly from GW (**Table 2**). The chlorophyll a:chlorophyll b ratio increased over time in C and GF, whereas in GW we observed a substantial decrease from 50 to 140 d.a.g. (**Table 2**). In contrast, carotenoids were more abundant at the beginning of summer (35 d.a.g.).

**Table 2:** Mean values (±SE) for chlorophyll content (Chl_**a**+**b**_), chlorophyll a:chlorophyll b ratio (Chl_**a**+**b**_) and carotenoids (Carot) 35, 50, and 140 days after girdling (d.a.g.) in control (C), fine girdled (GF), and wide girdled (GW) *Pinus canariensis*.

	**C**			**GF**			**GW**		
	35 d.a.g.	50 d.a.g.	140 d.a.g.	35 d.a.g.	50 d.a.g.	140 d.a.g.	35 d.a.g.	50 d.a.g.	140 d.a.g.
Chl_a+b_ (mg g^-1^)	1.68 ± 0.1^a^	1.67 ± 0.1^a^	1.56 ± 0.2^a^	1.32 ± 0.1^b^	1.50 ± 0.1^a^	1.34 ± 0.2^a^	1.23 ± 0.1^b^	0.95 ± 0.1^b^	0.83 ± 0.2^b^
Chl_a:b_	2.57 ± 0.2^a^	2.60 ± 0.1^a^	3.36 ± 0.5^a^	2.20 ± 0.2^ab^	2.21 ± 0.1^b^	2.67 ± 0.5^ab^	1.98 ± 0.1^b^	2.19 ± 0.1^b^	1.84 ± 0.4^b^
Carot (mg g^-1^)	0.28 ± 0.02^a^	0.30 ± 0.02^a^	0.28 ± 0.03^a^	0.21 ± 0.02^b^	0.25 ± 0.02^a^	0.22 ± 0.03^a^	0.20 ± 0.02^b^	0.19 ± 0.01^b^	0.08 ± 0.02^c^

Identical letters indicate homogeneous groups (*p* < 0.05).

**FIGURE 7:**
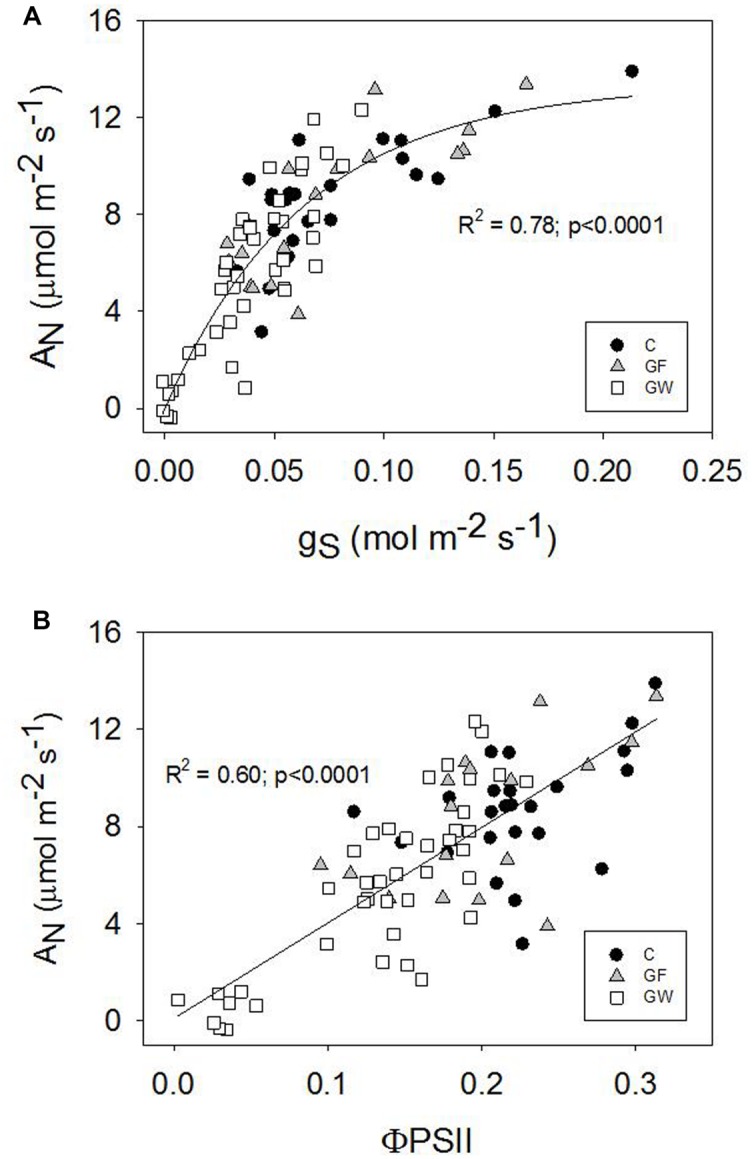
**(A)** Stomatal conductance (g_S_) vs. net photosynthetic rate (A_N_) and **(B)** operating quantum efficiency of photosystem II electron transport (ΦPSII) *vs.* net photosynthetic rate (A_N_), combining control and girdled plants.

A_N_ was strongly related to g_S_ and ΦPSII for all three treatments. The relationship was linear with ΦPSII (**Figure 7B**), whereas g_S_ and A_N_ responded coordinately at first but from g_S_ below 0.07 mol m^-2^ s^-1^, A_N_ decreased faster (**Figure 7A**). Variation of leaf temperature was similar in all treatments: 28.4 ± 0.5 C in September and 30.9 ± 0.7 C in the middle of June (**Figure 6D**).

### Hydraulic Conductivity and Wood Density

Girdling slightly increased wood density in both U and L and triggered a significant decrease in K_h_ in L and an increase in U, particularly in GW. K_S_ did not differ between segments or treatments but LSC was significantly higher in U in girdled plants (**Table 3**). Girdling did not cause xylem embolism in any of the samples. Maximum hydraulic conductance of the roots was affected by girdling and k_root_ in C was almost twice k_root_ in GF and GW (**Table 3**). Despite the reduction of leaf area of girdled plants during the first phase of the experiment (**Table 1**), LSc_root_ decreased in GF and GW by 28% (**Table 3**). k_root_ was positively correlated with maximum transpiration during the first phase of the experiment, measured 37 d.a.g. (*r* = 0.83) and ΦPSII (*r* = 0.72), but was negatively correlated with k_h_ (*r* = -0.55) and WD (*r* = -0.75) in U.

**Table 3:** Mean values (±SE) for wood density (WD), hydraulic conductance (k_h_), specific hydraulic conductivity (K_S_), leaf specific conductivity (LSC), root conductance (k_root_) and leaf specific root hydraulic conductance (LSc_root_) above (U) and below (L) the girdle 56 days after girdling (d.a.g.) in control (C), fine girdled (GF), and wide girdled (GW) *Pinus canariensis*.

	C		GF		GW	
	U	L	U	L	U	L
WD (g cm^-3^)	0.42 ± 0.02^c^	0.44 ± 0.02^bc^	0.44 ± 0.02^bc^	0.47 ± 0.02^ab^	0.45 ± 0.01^bc^	0.48 ± 0.01^a^
k_h_ (Kg s^-1^ MPa^-1^ × 10^-6^)	28.1 ± 6.1^ab^	43.2 ± 6.0^a^	33.7 ± 6.0^ab^	27.7 ± 6.2^ab^	41.0 ± 4.4^a^	27.2 ± 4.2^b^
K_S_ (Kg m^-1^ s^-1^ MPa^-1^)	0.66 ± 0.07^a^	0.71 ± 0.07^a^	0.67 ± 0.08^a^	0.71 ± 0.09^a^	0.79 ± 0.06^a^	0.66 ± 0.06^a^
LSC (Kg m^-1^ s^-1^ MPa^-1^ × 10^-6^)	56 ± 4^c^	85 ± 12^bc^	119 ± 14^a^	90 ± 10^ab^	120 ± 10^a^	78 ± 9^bc^
k_root_ (Kg s^-1^ MPa^-1^ × 10^-6^)	49.2 ± 7.1^a^		25.5 ± 7.9^b^		23.1 ± 5.0^b^	
LSc_root_ (Kg m^-2^ s^-1^ MPa^-1^ × 10^-6^)	98.5 ± 14.2^a^		70.5 ± 11.5^b^		67.1 ± 10.8^b^	

Identical letters indicate homogeneous groups (*p* < 0.05).

### Concentration of Abscisic Acid and Jasmonates

Abscisic acid content in C and GF was almost negligible, whereas in the xylem, upper phloem, and buds in GW we detected up to 33 nmol g^-1^ (**Table 4**). ABA-GE was abundant in the needles of plants in all treatments and also in buds in GW, where we found the highest content: 23.1 nmol g^-1^. No differences were found for JAs (**Table 4**).

**Table 4:** Mean concentration (±SE) of abscisic acid (ABA), abscisic acid glucose ester (ABA-GE), and jasmonic acid (JA) in xylem, needles, buds and phloem above (U) and below (L) the girdle 145 days after girdling (d.a.g.) in control (C), fine girdled (GF), and wide girdled (GW) *Pinus canariensis*.

	C			GF			GW		
	ABA (nmol g^-1^)	ABA-GE (nmol g^-1^)	JA (nmol g^-1^)	ABA (nmol g^-1^)	ABA-GE (nmol g^-1^)	JA (nmol g^-1^)	ABA (nmol g^-1^)	ABA-GE (nmol g^-1^)	JA (nmol g^-1^)
Buds	0.58 ± 0.2^c^	1.16 ± 0.6^c^	0.30 ± 0.1^ab^	0.28 ± 0.1^c^	0.40 ± 0.1^c^	0.18 ± 0.0^b^	16.40 ± 4.1^b^	23.11 ± 5.6^a^	0.41 ± 0.1^ab^
Needles	0.57 ± 0.1^c^	12.22 ± 0.2^b^	0.62 ± 0.2^ab^	0.61 ± 0.1^c^	12.78 ± 0.5^b^	0.44 ± 0.2^ab^	6.73 ± 2.2^b^	18.72 ± 4.9^ab^	0.21 ± 0.1^b^
Xylem	0.19 ± 0.0^c^	0.13 ± 0.0^c^	0.19 ± 0.0^b^	1.06 ± 0.7^c^	0.32 ± 0.1^c^	0.31 ± 0.1^ab^	32.76 ± 4.2^a^	0.61 ± 0.1^c^	0.36 ± 0.2^ab^
Phloem U	0.24 ± 0.1^c^	1.03 ± 0.3 ^c^	0.31 ± 0.1^ab^	0.39 ± 0.0^c^	1.46 ± 0.2^c^	0.26 ± 0.1^ab^	11.32 ± 4.0^b^	4.09 ± 1.4^b^	0.77 ± 0.3^a^
Phloem L	0.16 ± 0.0^c^	0.46 ± 0.1^c^	0.36 ± 0.1^ab^	0.19 ± 0.1^c^	0.52 ± 0.1^c^	0.23 ± 0.0^ab^	0.22 ± 0.2^c^	0.78 ± 0.2^c^	0.28 ± 0.1^ab^

Identical letters indicate homogeneous groups (*p* < 0.05) for each hormone and treatment.

## Discussion

### Sprouting and Cambial Growth

Girdling changed the carbon source–sink equilibrium in the trees. Roots no longer functioned as the sink of new assimilates, which tended to accumulate just above the girdle (Singh et al., 2003), triggering the increase in stem diameter above the girdle (zone U) due to irreversible structural growth and reversible swelling of living bark cells (De Schepper et al., 2010). In contrast, stem diameter growth ceased almost immediately just below the girdle (L), whereas in the base of the stem (B) it slowed down and girdled plants differed from controls only at the end of the experiment (**Figure 3**). Although cambial activity was directly affected by girdling (Chano et al., 2015), wood density barely changed. Only wood density of L in GW differed slightly from C (**Table 3**) but we could not discard that this change in density reflected changes in water content and thus green volume between C and GW plants. Wound tissue developed rapidly only from U (**Figure 2D**), and ca. 48 d.a.g. most GF plants had reconnected both sides of the girdle (**Figure 2C**) and the rate of diameter growth in U and L tended to balance out (**Figures 3A,B**).

Profuse sprouting was activated in L, indicating mobilization and consumption of stored carbohydrates in the roots as a response to serious above-ground damage to the plant (Del Tredici, 2001; Landhausser and Lieffers, 2002). Similar new basal shoots have been observed in other girdling studies with angiosperms (Wan et al., 2006; De Schepper et al., 2010). It appears that the available sugars were allocated to different purposes in U and L. Whereas the healing process and diameter growth were prioritized above the girdle (Chano et al., 2015), production of new photosynthetic material was the priority below the girdle as a consequence of blocked auxin transport (Wan et al., 2006). It may also have been due to higher concentrations of root-borne cytokinins, which promote the development of stem buds and shoots (Schmuelling, 2002). Profuse sprouting and reduced stem growth below the girdle supported a trade-off between cambial activity and sprouting (Figure 4) previously found in *Fagus sylvatica*, associated with local competition for carbohydrates between buds and close cambium (Collin et al., 2012). The interruption of sprout production approximately 30 d.a.g. in L and 60 d.a.g. in B may have indicated depletion of carbohydrates stored in the roots, as sprouting vigor is affected by the amount of the reserves in the roots (Zhu et al., 2012). When girdling was reversed, i.e., the phloem was reconnected, stem diameter growth below the girdle restarted but did not reach the same total increment of control trees (**Figure 3**).

### Water Use and Aboveground Biomass

The sharp decrease in whole plant transpiration 1 week after girdling onward may have been caused by foliage shedding or stomatal closure. Less aboveground dry mass and lower leaf area in girdled plants in the first harvest (56 d.a.g.) suggested that needle loss or less elongation of new needles could have partially triggered the reduction of plant transpiration as confirmed by negligible changes in leaf area specific transpiration. Decrease in g_S_ appeared to contribute more to reducing transpiration several weeks after girdling, as no drop in gs was detected 18 d.a.g. In the second measurement (35 d.a.g.), g_S_ had still not descended in larger plants, but in smaller girdled plants it decreased by up to 40% compared with controls. Later in the experiment (after summer), g_S_ and whole plant transpiration were almost negligible in GW, whereas in C and GF they increased, although leaf area specific transpiration did not fully recover in GF. Accumulation of carbohydrates in the needles of girdled trees is known to inhibit photosynthesis (Iglesias et al., 2002) and induce consequent stomatal closure. The significant increase in LMA in GW needles could be interpreted as indirect evidence of an increase in carbohydrate concentration (Day and DeJong, 1990; Domec and Pruyn, 2008). The reduction of g_S_ could also be attributed to the accumulation of ABA (Setter et al., 1980; Williams et al., 2000). We measured significant amounts of both ABA and its metabolite ABA-GE in needles, buds, xylem, and phloem above the girdle at the end of the experiment. We also expected GW plants to show higher levels of JA. However and despite the quick burst of JA in several species after mechanical injury (Wasternack, 2007) we did not find differences between treatments in any organ. Since we quantified hormones only at the end of the experiment, we could not discard the role of JA in stimulation of senescence and inhibiting root growth (Wasternack, 2007) in earlier stages, as previous works have reported that the signaling events mediated by this hormone occurs in a time window between a few minutes to several hours after wounding (León et al., 2001).

Abscisic acid, a well-known stress hormone, is produced in cells of stressed roots and may be delivered to the root apoplast and then transferred to the shoot via the transpiration stream (Sauter et al., 2002). It could have contributed to increased sink strength above the girdle (Yang et al., 2003) and to adjusting the physiological responses to wounding and decrease sink demand by regulating stomatal aperture (Setter et al., 1980). ABA synthesis is induced through the cleavage of carotenoid precursors (Nambara and Marion-Poll, 2005), thus lower values of water content and carotenoids in needles of GW plants 140 d.a.g. could also be the consequence of the increase in ABA in needles and play a role in leaf senescence (Schaper and Chacko, 1993; He and Jin, 1999). The reduction of leaf area after girdling was strengthened by the high levels of ABA found in buds which pointed to stronger bud dormancy in GW, preventing a new needle flush (Rinne et al., 1994). Soluble sugars above the girdle might have favored ABA-GE synthesis and accumulation in cell vacuoles (Sauter et al., 2002) regulating the ABA pool through glucosidase activity (Arve et al., 2013). Although the role of this metabolite is still unclear and it was initially reported as being physiologically inactive and functioning in storage and as a transport form of ABA (Jiang and Hartung, 2008), in *P. canariensis* free ABA was transported through xylem, and ABA-GE was found accumulated in significant amounts in needles of C, GF and GW plants, suggesting that this conjugate is trapped in the vacuoles and withdrawn from further metabolism (Sauter et al., 2002). In GW, the higher amount of ABA-GE in the phloem above the girdle than in the xylem or the phloem below the girdle suggested that leaves can release accumulated ABA-GE as a transport form to other tissues, such as buds, where β-glucosidase activity will release free active ABA (Yang and Zeevaart, 2006).

Girdling may affect sap transport in the xylem as a consequence of the concomitant reduction in leaf hydraulic conductance and stomatal closure (Domec and Pruyn, 2008; Sellin et al., 2013) and perhaps due to changes in the osmotic concentration of the xylem sap (Zwieniecki et al., 2004). In our experiment, we did not detect native xylem embolism in U or L or changes in K_S_. The reduction of leaf area in girdled trees with the increment of stem diameter in U resulted in higher efficiency of stem xylem in conducting water, measured as LSC, as occurs in dry-prone environments (López et al., 2013), but contrary to the reduction of stem hydraulic conductance capacity postulated in peach, a ring porous species, after girdling (Tombesi et al., 2014). In this sense, cambium removal early during the vegetative season affects less to tracheid bearing species than to ring porous species, dependent of the outermost part of the xylem to conduct water (Améglio et al., 2002). In contrast, the sharp decrease in root hydraulic conductance due to girdling reflected either changes in root development and morphology as a result of carbohydrate depletion or the inhibition of primary and lateral root elongation after the accumulation of cytokinins (Wan et al., 2006) caused by the decline in transpiration. Roots contain twice the concentration of starch early in the summer than later in the summer and therefore root respiration is less dependent on carbohydrate import in the early growing season (Högberg et al., 2001; Johnsen et al., 2007). Exhaustion of root reserves over time, intensified by the resprouting effort, and the suberization observed during stress, reduce root permeability (Steudle, 2000). In the same way, a reduction in nutrients decreases root conductance, as aquaporin activity is affected (Carvajal et al., 1996; Vandeleur et al., 2014). We found that, to a large degree, root conductance governs the maximum rate of transpiration that the plant is able to sustain. Root starvation after photosynthate withdrawal could therefore have decisively altered plant–water relations. The long-term response to the decrease in root hydraulic conductance would entail a lower moisture content of the stem and the progressive loss of parenchyma viability (Taylor and Cooper, 2002). Leaves of girdled plants were supplied with less water, measured as LSc_root_, and although transpiration was almost negligible at the end of the experiment in GW, leaf RWC was significantly lower in GW than in C and GF.

### Gas Exchange and Damage to the Photosynthetic Apparatus

Downregulation of photosynthesis after girdling was consistent with other studies showing a negative feedback effect of carbohydrate accumulation on A_N_ (DeJong, 1986; Day and DeJong, 1990; Myers et al., 1999; Iglesias et al., 2002; Urban and Alphonsout, 2007; Vemmos et al., 2012). The more rapid decline in A_N_ than in g_S_ from a g_S_ lower than *ca.* 0.10 mol m^-2^ s^-1^ in *P. canariensis* (**Figure 7A**), and similar results observed in hybrid aspen (Sellin et al., 2013) and mango (Urban and Alphonsout, 2007), were attributed to the depressing effect of girdling on photosynthesis, primarily because of changes in the electron transport rate rather than changes in g_S_ (Urban and Alphonsout, 2007). Two likely explanations previously proposed for this negative feedback on photosynthesis were an excess of starch grains leading to physical damage to thylakoids and the subsequent decrease of chlorophyll levels (Schaffer et al., 1986), and inhibition of photosynthetic genes regulated by carbohydrate content (Paul and Foyer, 2001). Girdling changed the concentration and ratio of photosynthetic pigments. Chlorophylls progressively decreased in girdled plants and we observed chlorosis in mature leaves early in the experiment. The effect was rapidly reversed when the phloem reconnected and only the chl_a:b_ ratio took longer to recover to the levels of control plants.

Actual efficiency of PSII, ΦPSII was affected by sink limitation earlier than by maximum photochemical efficiency (F_V_/F_M_). The reduction of ΦPSII pointed to a decrease of absorbed energy and also to loss of efficiency in energy processing. The chl_a:b_ ratio has been correlated with the ratio of PSII cores to light harvesting-protein complex (LHCII), in which the majority of chl_b_ is located (Kitajima and Hogan, 2003). In girdled plants the chl_a:b_ ratio decreased suggesting a faster drop in light processing than light harvesting. Reduced energy utilization by CO_2_ assimilation, in combination with high energy capture, is potentially harmful and can result in over-reduction of the electron transport chain, photoinhibition and oxidative stress (Badger, 1985). The decreased observed in A_N_ was correlated with a decrease in ΦPSII and an increase in harmless non-radiative energy dissipation that provided photoprotection from oxidative damage following sink limitation (Myers et al., 1999; Adams et al., 2005). As a result, F_V_/F_M_ was higher than the optimum 0.83 (Maxwell and Johnson, 2000) in 61% of girdled plants 35 d.a.g. Partial recovery after reconnection of the phloem, with most plants showing F_V_/F_M_ higher than 0.8 in our experiment, and earlier results after flowering (new sinks for assimilates) in mango trees, support the idea that the photosynthetic system was maintained in a highly photoprotected state in girdled branches or stems (Urban and Alphonsout, 2007).

## Conclusion

Girdling affected both the downward carbon flow and the upward water flow. Blocking the root sink led to an increase in the increment in diameter growth above the girdle and the decrease below it, accompanied by profuse sprouting below the girdle that stopped after depletion of carbohydrates stored in the roots. Removing the sprouts may have jeopardized the plant strategy to recover from the damage, although the adaptation of this species to wounding allowed plants to survive more than 140 days without carbohydrate supply to the roots. The accumulation of carbohydrates in the aerial parts caused the reduction of whole plant transpiration due to leaf shedding and stomata closure due to feedback inhibition of photosynthesis and higher ABA and ABA-GE metabolism. Girdling strongly decreased root hydraulic conductance, whereas the water-conducting capacity of the stem increased above the girdle. Both ΦPSII and F_V_/F_M_ decreased in girdled plants in comparison with controls. F_V_/F_M_ values did not recover after wound healing, suggesting that phloem reconnection may still not be fully functional in some plants at the end of the experiment.

## Author Contributions

LG provided the idea after observations in natural populations. RL, PP and LG designed the experiment. RL and PP performed the wounds and carried out measurements of growth, gas exchange, fluorescence and plant hydraulics. RB analyzed the hormones. All authors contributed to interpret the results. RL drafted the manuscript. All authors read and approved the final manuscript.

## Conflict of Interest Statement

The authors declare that the research was conducted in the absence of any commercial or financial relationships that could be construed as a potential conflict of interest.
